# Coronal Sealing Ability of Three Temporary Filling Materials

**Published:** 2012-03-01

**Authors:** Mandana Naseri, Zohreh Ahangari, Mahyar Shahbazi Moghadam, Manijeh Mohammadian

**Affiliations:** 1. Department of Endodontics, Dental School, Shahid Beheshti University of Medical Sciences, Tehran, Iran; 2. Department of Operative Dentistry, Dental Branch, Islamic Azad University of Medical Sciences, Tehran, Iran; 3. General Dentist, Tehran, Iran

**Keywords:** Dental Leakage, Methylene Blue, Temporary Filling, Time

## Abstract

**Introduction:**

Providing adequate coronal seal of temporary filling materials is critical for the success of root canal therapy. The aim of this in vitro study was to compare coronal seal ability of three restorative materials over different periods of time.

**Materials and Methods:**

Ninety-eight molar teeth were selected. Once access cavities were prepared, teeth were divided randomly into three time groups (1 day, 1 week, and 4 weeks). Each group was then subdivided into three groups of 10 teeth. Each subgroup was restored using one of three restorative materials including Coltosol, Cavizol and Zonalin and then incubated in distilled water at 37ºC. The samples were then immersed in 2% methylene blue dye. After rinsing and drying, teeth were sectioned longitudinally and examined for dye penetration. Kruskal-Wallis and Mann Whitney U tests were used to analyze the data.

**Results:**

All experimented materials showed increasing leakage from the 1st day to the 4th week. Zonalin showed more leakage than Coltosol and Cavizol at each time interval (P<0.05), but there was no significant difference between Coltosol and Cavizol.

**Conclusion:**

Coltosol and Cavizol are suitable temporary materials for up to 1 week.

## Introduction

Bacterial infection has been declared as the most common cause of the pulpal and periradicular diseases [1][2]. Therefore; the major goals of root canal treatment are the chemo mechanical debridement and sealing of the root canal system to eliminate the irritants. Previous studies have demonstrated that coronal leakage can compromise the success of root canal therapy [3][4][5] and the quality of the coronal seal is just as important as the quality of the root canal filling for periapical health, as shown by Ray and Trope who demonstrated correlation between radiologically inappropriate coronal restoration and the poor periapical status of root-filled teeth [6]. Torabinejad et al. showed that bacteria were able to pass along root filling within 5 to 73 days from the coronal to the apical end [7]. Extensive leakage upon unfilled cavity is shown by Khayat et al. [8]. They used fresh human saliva to show that root filling were entirely penetrated by microorganisms of saliva within 48 days, in unsealed teeth.

Also, lack of satisfactory temporary restorations during endodontic therapy is stated as second contributing factors in continuing pain after commencement of treatment [9]. Accordingly, temporary filling materials must provide an adequate seal against ingress of bacteria, fluids and other debris from the oral cavity to the root canal system, and at the same time prevent seepage of intracanal medicaments.

Many studies have shown contradictory results of sealing ability of different temporary restorative materials, which might be attributed to the different methodologies used in these studies, especially with respect to techniques used to measure coronal microleakage over different periods of time [10][11][12].

The aim of this in vitro study was to compare the sealing properties of Coltosol, Cavizol and Zonalin used as temporary filling material in coronal access openings at different time intervals.

## Materials and Methods

Ninety-eight caries free extracted molar teeth were randomly selected for this experimental study. After cleaning soft tissues and calculus, the teeth were stored in 0.9% saline and kept moist at all time throughout this experiment. They were divided into three experimental groups of 30 teeth and 2 control groups of four teeth each. Standard endodontic access cavities of approximately 4×4 mm wide upon measuring by periodontal probe were prepared through the occlusal surface, using a high-speed air turbine handpiece under water coolant with a no.4 round bur for initial and a diamond fissure bur to extend the preparation. The pulp tissue was removed and the teeth were irrigated with 5.25% sodium hypochlorite. Drying the chambers, a small cotton pellet was packed on the floor of chamber in a manner that the depth of the opening, measured by periodontal probe, could accommodate 4 mm thickness of temporary material.

Each experimental group was divided into three subgroups including 10 teeth each, which were filled by one of experimental materials including Cavizol (Golchai, Tehran, Iran), Coltosol (Coltene, Altstatten, Switzerland) and Zonalin (Kemdent, Wiltshire, UK) by the same operator. Zonalin was mixed in a liquid to powder ratio recommended by the manufacture.

Access cavities were prepared but no filling was placed in positive control group except a small cotton pellet on the floor of the chamber, in contrast with the negative group which received no access preparation.

All specimens were stored at 37°C and 100% humidity for 1 day to ensure complete setting of the temporary filling materials. Then all the experimental and positive control groups were covered with two layers of nail varnish leaving 1 mm around the access cavity margins. The apical foramina were sealed with red wax.

All teeth were immersed in 2% methylene blue dye according to the test time; one day, one week and 4 weeks for the first, second and third experimental groups, respectively. Control groups were placed in dye for 4 weeks. At the end of experimental time, removing the teeth from dye, they were thoroughly rinsed in tap water for 2 hours, longitudinally sectioned in a buccolingually direction using a carbide fissure bur number 4 with copious water cooling. After using the bur to make a cut in the crown toward the pulp chamber, the teeth were broken by wedging a plastic instrument. All the samples were blindly examined by the same operator and the greater depth of methylene blue dye penetration was measured in millimeter at both sides of the specimen as an indicator of coronal microleakage by using a loupe at ×3 magnification (Table 1).

**Table 1:** Scoring of dye leakage

**Degree of leakage**	**Depth of penetration**
0	No staining
1	Staining up to 1 mm
2	Staining up to 2 mm
3	Staining up to 3 mm
4	Staining up to 4 mm
5	Staining more than 4 mm

Data were analyzed using Kruskal-Wallis and Mann-Whitney U tests. The level of significance was set at P<0.05.

## Results

Dye penetration was observed in all experimental materials. Positive control group demonstrated complete dye penetration, and negative controls remained leakage-free up to 4th week. The dye penetration scores for each material based upon the time are given in (Table 2). Zonalin showed leakage score ≥ 3, during the experiment time.

**Table 2:** Dye penetration scores of experimental materials at different times

**Dye score**	**Coltosol**			**Cavizol**			**Zonalin**		
	1d	1w	4ws	1d	1w	4ws	1d	1w	4ws
**0**	6	0	0	6	0	0	0	0	0
**1**	2	2	1	1	2	0	0	0	0
**2**	0	4	5	1	4	3	0	0	0
**3**	2	4	2	2	4	6	4	1	0
**4**	0	4	2	0	0	1	3	6	2
**5**	0	0	0	0	0	0	3	3	8
**Total number**	10	10	10	10	10	10	10	10	10

Kruskal-Wallis test (Table 3) showed significant differences between microleakage in Zonalin and two other materials at each time interval, with Zonalin showing more leakage (P<0.05).

**Table 3:** Mean values and Standard Deviation (SD) (mm) of coronal microleakage of materials tested at three time periods

**Time**	**Material**	**N**	**Min (score)**	**Max (score)**	**Mean (SD)**	
**1 day**	Coltosol	10	0	3	0.8 (1.22)	*P*=0.0001
	Cavizol	10	0	3	0.9 (1.28)	
	Zonalin	10	3	5	3.9 (0.87)	
**1 week**	Coltosol	10	1	3	2.2 (0.79)	*P*=0.0002
	Cavizol	10	1	4	2.4 (1.07)	
	Zonalin	10	3	5	4.2 (0.63)	
**4 weeks**	Coltosol	10	1	4	2.5 (0.97)	*P*=0.0001
	Cavizol	10	2	4	2.8 (0.63)	
	Zonalin	10	4	5	4.8 (0.42)	

There was no significant difference between Coltosol and Cavizol at all intervals (P=0.94, 0.57 and 0.93 for 1 day, 1 and 4 weeks, respectively).

Totally, leakage of all experimented materials showed increase from the first day to one week which was statistically significant (P<0.05), but there was no significant difference between the data belonged to 1st and 4th weeks microleakage in Coltosol and Cavizol (P=0.47and 0.36, respectively). No significant difference was achieved among the three experimental time for Zonalin group (P<0.05).

## Discussion

After obturation of the root canal system, the occlusal access cavity should be properly sealed [13]. It has been shown that the prognosis of root-filled teeth can be improved by efficient coronal seal [5][14][15].

Although, this was an in vitro study, the usage of all restorative materials followed normal clinical placement procedure by one operator to reduce the chance of manipulative variable. Moreover, all specimens were incubated at 37°C to simulate clinical condition. Inserting a 4 mm thickness of temporary material was based on the report of Webber et al.[16] and Zmener et al. [10] suggesting that at least 3.5-4 mm thickness of material is needed to provide a good coronal seal.

The rationale for testing the four materials after 1 day, 1 and 4 weeks separately was that these are frequently used time intervals in dental practice either between endodontic treatment appointments or after obturation and before the placement of permanent restoration.

Several studies have been performed to examine the sealing efficacy of temporary filling materials by using dye as a simple easy and accurate method [11][12][17]. Although the molecular size of dyes such as methylene blue is smaller than bacteria, so may be a tool for comparing relative leakage, but it doesn’t simulate the types of microbial leakage that may occur clinically.

As thermo cycling may affect microleakage of some temporary filling materials specially Zonalin [11][12], this was not performed in this study.

Zonalin is a temporary filling material that requires mixing the powder and liquid before using. It is a polymer-modified ZOE, reinforced with 20-40% by weight polymethyl methacrylate [18]. Adding this polymer allows the material to be relatively hydrophobic, thus maintaining its integrity for prolonged periods [19][20]. The results of the present study indicated that at any given time, Zonalin has significantly higher leakage scores compared with Cavizol and Coltosol, but there is no significant difference between two later. Similar results have shown by other investigators [17][21][22][23]. This result can be due to the fact that powder and liquid have to be mixed together which may be the cause of reduced homogeneity. Deveaux et al. [24] and Madarati et al. [12] showed numerous voids on surfaces of IRM samples, a temporary filling material just as Zonalin. Also, Zmener reported that some IRM specimens not only leaked at the dentin restorative interface, but also absorbed the dye into the bulk of the material [10].

Cavizol and Coltosol are from Cavit category which expands in contact with moisture. This expansion provides good adaptation between the restorative material and cavity walls. On the other hand, they are premixed, ready-to-use and quick to be placed and adjusted in the access cavity. These good manipulation properties are considered as being supplementary factors for good coronal seal ability [12] [25][26][27].Similar results, showing less seal ability of IRM than Cavit and Coltosol, have been reported by other investigators [10][11][17][21][28].

The results of this study showed that although there was a significant difference between the mean coronal micro leakage at one day and 1 or 4 weeks, but no difference between two later periods for Cavizol and Coltosol, in contrast with Zonalin group which showed no differences at three time periods. So within the conditions of this study, it can be stated that microleakage of each material increased by the time from one day to 4 weeks, but Cavizol and Coltosol demonstrated a good sealing ability up to one week which was statistically different from that demonstrated by Zonalin which showed poor sealing ability even at first day.

## Conclusions

The results of this in vitro study are not clinical evidence; however they suggest that temporary fillings be placed for shortest possible period of time. Ideally, a definitive coronal restoration should be placed as soon as possible after root canal treatment.

## References

[1] Kakehashi S, Stanley HR, Fitzgerald RJ (1966). The effects of surgical exposures of dental pulps in germfree and conventional laboratory rats. J South Calif Dent Assoc.

[2] Moller AJ, Fabricius L, Dahlen G, Ohman AE, Heyden G (1981). Influence on periapical tissues of indigenous oral bacteria and necrotic pulp tissue in monkeys. Scand J Dent Res.

[3] Saunders WP, Saunders EM (1994). Coronal leakage as a cause of failure in root-canal therapy: a review. Endod Dent Traumatol.

[4] Tronstad L, Asbjornsen K, Doving L, Pedersen I, Eriksen HM (2000). Influence of coronal restorations on the periapical health of endodontically treated teeth. Endod Dent Traumatol.

[5] Heling I, Gorfil C, Slutzky H, Kopolovic K, Zalkind M, Slutzky-Goldberg I (2002). Endodontic failure caused by inadequate restorative procedures: review and treatment recommendations. J Prosthet Dent.

[6] Ray HA, Trope M (1995). Periapical status of endodontically treated teeth in relation to the technical quality of the root filling and the coronal restoration. Int Endod J.

[7] Torabinejad M, Ung B, Kettering JD (1990). In vitro bacterial penetration of coronally unsealed endodontically treated teeth. J Endod.

[8] Khayat A, Lee SJ, Torabinejad M (1993). Human saliva penetration of coronally unsealed obturated root canals. J Endod.

[9] Abbott PV (1994). Factors associated with continuing pain in endodontics. Aust Dent J.

[10] Zmener O, Banegas G, Pameijer CH (2004). Coronal microleakage of three temporary restorative materials: an in vitro study. J Endod.

[11] Aminozarbian MG, Feizianfard M, Karimi M (2009). Sealing ability of three temporary filling materials in endodontically-treated teeth. Iran Endod J.

[12] Madarati A, Rekab MS, Watts DC, Qualtrough A (2008). Time-dependence of coronal seal of temporary materials used in endodontics. Aust Endod J.

[13] Shahi S, Samiei RS, Nezami H (2010). In vitro comparison of dye penetration through four temporary restorative materials. Iran Endod J.

[14] Safavi KE, Dowden WE, Langeland K (1987). Influence of delayed coronal permanent restoration on endodontic prognosis. Endod Dent Traumatol.

[15] Asgary S, Shadman B, Ghalamkarpour Z, Shahravan A, Ghoddusi J, Bagherpour A, Akbarzadeh Baghban A (2010). Periapical status and quality of root canal fillings and coronal restorations in Iranian population. Iran Endod J.

[16] Webber RT, del Rio CE, Brady JM, Segall RO (1978). Sealing quality of a temporary filling material. Oral Surg Oral Med Oral Pathol Oral Radiol Endod.

[17] Tewari S (2002). Assessment of coronal microleakage in intermediately restored endodontic access cavities. Oral Surg Oral Med Oral Pathol Oral Radiol Endod.

[18] Manappallil JJ (2003). Basic Denial Materials, 3th Edition.

[19] Koagel SO, Mines P, Apicella M, Sweet M (2008). In vitro study to compare the coronal microleakage of Tempit UltraF, Tempit, IRM, and Cavit by using the fluid transport model. J Endod.

[20] Jensen AL, Abbott PV (2007). Experimental model: dye penetration of extensive interim restorations used during endodontic treatment while under load in a multiple axis chewing simulator. J Endod.

[21] Ciftci A, Vardarli DA, Sonmez IS (2009). Coronal microleakage of four endodontic temporary restorative materials: an in vitro study. OralSurg Oral Med Oral Pathol Oral Radiol Endod.

[22] Balto H (2002). An assessment of microbial coronal leakage of temporary filling materials in endodontically treated teeth. J Endod.

[23] Lai YY, Pai L, Chen CP (2007). Marginal leakage of different temporary restorations in standardized complex endodontic access preparations. J Endod.

[24] Deveaux E, Hildelbert P, Neut C, Romond C (1999). Bacterial microleakage of Cavit, IRM, TERM, and Fermit: a 21-day in vitro study. J Endod.

[25] Chohayeb AA, Bassiouny MA (1985). Sealing ability of intermediate restoratives used in endodontics. J Endod.

[26] Uctasli MB, Tinaz AC (2000). Microleakage of different types of temporary restorative materials used in endodontics. J Oral Sci.

[27] Aledrissy HII, Abubakr NH, Ahmed N (2011). Coronal microleakage for readymade and hand mixed temporary filling materials. Iran Endod J.

[28] Veloso HH, Estrela CRA, Decurcio DA, Alves D, Estrela C (2008). Microbial microleakage in temporary restorative materials after post space preparation. Revista Odonto Ciência (Journal of Dental Science).

